# Associations between lamb survival and prion protein genotype: analysis of data for ten sheep breeds in Great Britain

**DOI:** 10.1186/1746-6148-5-3

**Published:** 2009-01-21

**Authors:** Simon Gubbins, Charlotte J Cook, Kieran Hyder, Kay Boulton, Carol Davis, Eurion Thomas, Will Haresign, Stephen C Bishop, Beatriz Villanueva, Rachel D Eglin

**Affiliations:** 1 Institute for Animal Health, Pirbright Laboratory, Ash Road, Pirbright, Surrey, GU24 0NF, UK; 2 Centre for Epidemiology and Risk Analysis, Veterinary Laboratories Agency, Woodham Lane, New Haw, Addlestone, Surrey, KT15 3NB, UK; 3 Meat and Livestock Commission, Winterhill House, Snowdon Drive, Milton Keynes, MK6 1AX, UK; 4 Innovis Ltd, Peithyll Centre, Capel Dewi, Aberystwyth, Ceredigon, SY23 3HU, UK; 5 Institute of Biological, Environmental and Rural Sciences, Aberystwyth University, Llanbadarn Campus, Aberystwyth, Ceredigon, SY23 3AL, UK; 6 The Roslin Institute and R(D)SVS, University of Edinburgh, Roslin BioCentre, Roslin, Midlothian, EH25 9PS, UK; 7 Scottish Agricultural College, West Mains Road, Edinburgh, EH9 3JG, UK

## Abstract

**Background:**

Selective breeding programmes, based on prion protein (PrP) genotype, have been introduced throughout the European Union to reduce the risk of sheep transmissible spongiform encephalopathies (TSEs). These programmes could have negative consequences on other important traits, such as fitness and production traits, if the PrP gene has pleiotropic effects or is in linkage disequilibrium with genes affecting these traits. This paper presents the results of an investigation into associations between lamb survival and PrP genotype in ten mainstream sheep breeds in Great Britain (GB). In addition, the reasons for lamb deaths were examined in order to identify any associations between these and PrP genotype.

**Results:**

Survival times from birth to weaning were analysed for over 38000 lambs (2427 dead and 36096 live lambs) from 128 flocks using Cox proportional hazard models for each breed, including additive animal genetic effects. No significant associations between PrP genotype and lamb survival were identified, except in the Charollais breed for which there was a higher risk of mortality in lambs of the ARR/VRQ genotype compared with those of the ARR/ARR genotype. Significant effects of birth weight, litter size, sex, age of dam and year of birth on survival were also identified. For all breeds the reasons for death changed significantly with age; however, no significant associations between reason for death and PrP genotype were found for any of the breeds.

**Conclusion:**

This study found no evidence to suggest that a selective breeding programme based on PrP genotype will have a detrimental effect on lamb survival. The only significant effect of PrP genotype identified was likely to be of little consequence because an increased risk of mortality was associated with a genotype that is selected against in current breeding strategies.

## Background

Large-scale selective breeding programmes have been introduced throughout the European Union to reduce the risk of sheep transmissible spongiform encephalopathies (TSEs) and, in particular, the risk to human health posed by the possible presence of bovine spongiform encephalopathy (BSE) in sheep [1]. These programmes exploit a strong host genetic component at the ovine prion protein (PrP) gene, which influences both the risk of infection and the incubation period for scrapie [2-4]. They aim to decrease the frequency of high risk alleles (ARQ and VRQ) and increase the frequency of the major low risk allele (ARR).

Concerns have been expressed, however, that such large-scale breeding programmes based on one specific factor (in this case, PrP genotype) could have detrimental side-effects on sheep performance. These concerns stem from farmers' assertions that sheep carrying high-risk genotypes are more hardy or perform better than their flock-mates which carry low-risk genotypes [5,6], and from the hypothesis that the high-risk VRQ allele is maintained in the sheep population because it confers a selective advantage in a scrapie-free environment [7]. Such a situation could arise if the PrP gene has a pleiotropic effect on, or is in linkage disequilibrium with, production or survival traits.

To date there have been numerous studies examining associations between production traits and PrP genotype [8-14], none of which has found any consistent or clear relationships. Less attention has been given to investigating associations between survival traits and PrP genotype. One study found that animals of susceptible PrP genotypes had a shorter life expectancy in scrapie-affected flocks, even if they did not succumb to clinical disease [15]. More recently, a study of survival in lambs of the Scottish Blackface breed found that animals carrying the ARQ allele had higher postnatal survival than lambs carrying the ARR or AHQ alleles, both of which are associated with a lower risk of clinical disease [16].

In this paper, we report the results of an investigation into associations between lamb survival and PrP genotype in ten mainstream sheep breeds in Great Britain (GB). These breeds were drawn from all sectors of the British sheep industry, from hardy hill breeds to those raised in less hostile environments, and include some of the most important commercial breeds. The aim of the study was not to estimate or compare absolute lamb mortality in the different breeds, rather it was to identify factors influencing relative mortality within individual breeds, in particular, PrP genotype. In addition to lamb survival, we also examined the reason for lamb deaths in order to identify any associations between these and PrP genotype.

## Methods

### Breeds included in study

The ten sheep breeds included in the study (Table 1) were selected to cover the different sectors of the British sheep industry (see, for example, [17]). The hill breeds (Beulah Speckled Face, Scottish Blackface, North Country Cheviot, Welsh Mountain), which are kept in the harsh hill environment, comprise four of the five most numerous purebreeds and account for 27.5% of ewes kept in GB. The North Country Cheviot (NCC) breed was subdivided into Hill and Park types because of differences in terms of proflicacy, size and the environment in which each type is kept. (The Park type produces more twins, tends to be larger and is raised in a less extreme environment than the Hill type.) The Bluefaced Leicester is the most numerous breed in the longwool sector, and is the predominant sire breed of the crossbred ewe population. Terminal sire rams produce a high proportion of lambs for slaughter; the Texel and the Charollais are two of the three most important breeds in this sector, contributing 24.4% and 7.6% of all rams in GB, respectively. Finally, the Lleyn and the Poll Dorset breeds represent two of the three most numerous breeds in the self-contained sector (breeds managed in stand-alone flocks that have developed in a less-extreme environment than the hill breeds) and account for 1.6% and 0.6% of purebred ewes kept in GB, respectively.

**Table 1:** Breeds, industry sector and the number of lambs and flocks included in the study.

		no. lambs (no. lambs with birth weight recorded)		
			
breed	industry sector	live	dead	no. flocks collecting records
Beulah Speckled Face	hill	1735 (1253)	71 (44)	7
Bluefaced Leicester	longwool	678 (587)	85 (66)	5
Charollais	terminal sire	5721 (4032)	520 (402)	19
Lleyn	self-contained	3475 (2927)	178 (153)	9
Poll Dorset	self-contained	4491 (3149)	215 (177)	9
North Country Cheviot (Hill)	hill	2030 (968)	158 (82)	11
North Country Cheviot (Park)	hill	2255 (1427)	222 (129)	13
Scottish Blackface	hill	4098 (2201)	479 (312)	17
Texel	terminal sire	9293 (6771)	401 (259)	29
Welsh Mountain	hill	2288 (1475)	98 (66)	9

Total	-	36096 (24818)	2427 (1690)	128

### Farmer recruitment

Farmers were eligible to join the study if they recorded their flock performance traits using the Meat and Livestock Commission's Signet Sheepbreeder, a genetic evaluation programme, and were members of the Ram Genotyping Scheme (RGS) of the National Scrapie Plan for GB (NSP). All eligible breeders were invited to join the study by letter from Signet. In addition, the study was promoted amongst farmers by telephone and direct contact at breeder meetings. Farmers provided flock details and signed consent forms to release their Signet and NSP data to research partners in the study.

### PrP genotyping

Tissue samples were taken from dead lambs found by the farmers using Tipyfix^® ^ear tags (Agribiogen Biotechnologie GmbH). Samples were sent for PrP genotyping using proprietary commercial technology to Cellmark, part of Orchid Biosciences Europe Ltd, under contract to Innovis Ltd (formerly CBS Technologies). Results were collated by Innovis Ltd and provided to the Veterinary Laboratories Agency (VLA). Live lambs were blood sampled by Animal Health (formerly State Veterinary Service) as part of the NSP on special visits to participating flocks each year. Blood samples were processed and PrP genotyped using proprietary commercial technology by Cellmark (70%) or LGC (30%). All live lambs on farm were sampled, with visits encouraged to take place before replacement lambs had been selected for use in the flock and before lambs were sent for sale or slaughter.

Full details of the PrP genotypes included in the study for each breed are presented in the additional material (see additional file 1). The data-sets for most breeds included at least 50% of the PrP genotypes commonly found in the breed; those not present in the data-sets tended to be the higher-risk, VRQ-bearing genotypes.

### Data sets

Lambs from cohorts born in 2004, 2005 and 2006 were included in the study. Signet provided recorded data on dam, sire, birth weight (where available), litter size born, litter size reared and fostering information for all lambs born in participating flocks. Genotype data for live animals were provided to Signet by the NSP Administration Centre following blood sampling visits to each farm. The genotypes were matched to Signet data using the ear tag provided on the day of sampling. These data were subsequently provided to the VLA via a regular electronic transfer of animal data each year.

Data on dead lambs were collected using a submission form, which was linked by barcode to the tissue sample sent for PrP genotyping. Data captured on the form included ear tag number, date of birth, date of death, dam identity, sex and the breeder's opinion of the reason for death. Submission forms were sent to VLA, where the data were double-entered onto a database. The results of the tissue genotyping were returned to Innovis by Cellmark and provided electronically to the VLA, where the PrP genotype was matched by barcode to individual animals.

The number of records in each data-set ranged from around 800 lambs for the Bluefaced Leicester to around 10000 lambs for the Texel, and the number of dead lambs varied from 71 for the Beulah Speckled Face to 520 for the Charollais (Table 1).

### Statistical analysis

Lamb survival times were analysed from birth (0 days) to weaning (120 days). Weaning was chosen as the end point, because this is an age before which lambs are dispersed, for example, selected as replacements or sent for slaughter or sale. A total of 204 lambs in the data-set died after 120 days, and these were included in the analyses as censored at 120 days. Survival times were modelled using Cox proportional hazard models [18], with the baseline hazard stratified by flock and including additive animal genetic effects. Pedigrees were constructed using only the dam and sire information for each lamb. Exploratory analysis produced estimates for the animal-level variance (σ^2^) in the range 0.2 to 0.4 for three breeds (Bluefaced Leicester, Scottish Blackface and Texel); however, the variance estimate was close to zero (<0.01) for the remaining seven breeds. This is a consequence of the relatively small number of mortalities available in several of the breeds and the fact that lamb survival is a lowly heritable trait, particularly if only a subsample of all mortalities are recorded. Consequently, the animal-level variance was fixed at σ^2 ^= 0.2 for all breeds rather than being estimated; this is similar to estimates obtained in previous studies of lamb survival [19-21].

Survival data were analysed separately for each breed. Two models were constructed: one including birth weight as a fixed effect and one excluding birth weight. In each case model selection proceeded by stepwise deletion of non-significant (*P *> 0.05) terms, starting from a model incorporating PrP genotype (see additional file 1, for details of the PrP genotypes included in the analysis for each breed), litter size (1/2/≥ 3), sex, age of dam (≤ 2/3/4/5/≥ 6 years old) and year of birth as fixed effects, and birth weight as a covariate with both linear and quadratic terms, when included; animals with missing values for any of these factors were excluded from the analysis. This was a particular issue for birth weight, where an appreciable number of records were missing data on this factor (Table 1), ranging from 14% of records for the Bluefaced Leicester to 52% for the North Country Cheviot (Hill). The models were implemented in R [22] using the kinship package [23].

The reason for death supplied by the breeder was coded into one of five classes: lambing-associated (e.g. stillborn, malpresentation, enclosed in amniotic sac); misfortune (e.g. taken by predator, drowned); physical (e.g. mineral deficiency); disease; or other, including unknown. Potential associations were explored between reason for death and PrP genotype, breed, sex, age at death (coded as: birth (0 days); perinatal period (1–14 days); perinatal period to weaning (15–120 days); post-weaning (≥ 120 days)) and year of birth. Initially, a multinomial log-linear model was constructed including all the above five factors, together with pairwise interactions between breed and the four remaining factors. Model simplification proceeded by stepwise deletion of non-significant terms (*P *> 0.05). The models were implemented in R [22] using the nnet package [24].

## Results

### Survival times

The final models for each breed are presented in the additional material (see additional file 1); a summary of the results is presented in Table 2. For all but one of the breeds, there were no significant associations between PrP genotype and lamb survival (Table 2). There was, however, a significant association in the Charollais breed, for which the hazard of death in ARR/VRQ lambs was 2.7 times greater than that for ARR/ARR lambs (hazard ratio (HR): 2.67; 95% confidence limits (CI): 1.31–5.44). There was no significant difference in survival between ARR/ARQ lambs (the only other genotype reported in the sampled population for this breed) and ARR/ARR lambs (HR: 0.90; 95% CI: 0.71–1.15). This effect was only apparent when birth weight was not included in the analysis, because none of the dead ARR/VRQ lambs had a birth weight recorded. A similar, but non-significant, increased risk of mortality in ARR/VRQ compared to ARR/ARR lambs was also observed for the Lleyn breed; however, this was not seen for any other breeds in the study. Indeed, there were no consistent patterns in the effects of PrP genotype on lamb survival across breeds, whether statistically significant or not.

**Table 2:** Summary of factors influencing lamb survival times in ten sheep breeds in GB.

	factor^†^					
	
Breed	PrP genotype	birth weight^‡^	litter size	sex	age of dam	year of birth
*model including birth weight*						
Beulah Speckled Face	ns	***^a^	ns	***	ns	**
Bluefaced Leicester	ns	**^a^	ns	ns	ns	***
Charollais	ns	***^b^	**	ns	ns	***
Lleyn	ns	***^a^	ns	***	ns	***
North Country Cheviot (Hill)	ns	***^a^	*	***	ns	***
North Country Cheviot (Park)	ns	**^b^	ns	***	ns	***
Poll Dorset	ns	***^b^	*	***	ns	***
Scottish Blackface	ns	***^b^	*	***	**	***
Texel	ns	***^a^	ns	***	*	ns
Welsh Mountain	ns	***^a^	ns	***	**	**
*model excluding birth weight*						
Beulah Speckled Face	ns	-	***	***	ns	ns
Bluefaced Leicester	ns	-	ns	ns	*	**
Charollais	*	-	***	ns	ns	***
Lleyn	ns	-	*	***	**	***
North Country Cheviot (Hill)	ns	-	***	***	ns	***
North Country Cheviot (Park)	ns	-	***	***	***	ns
Poll Dorset	ns	-	***	***	**	***
Scottish Blackface	ns	-	***	***	***	***
Texel	ns	-	**	***	**	**
Welsh Mountain	ns	-	**	***	***	ns

^† ^significance in final model or when deleted from the model: ns (*P *> 0.05); * (0.01 <*P *≤ 0.05); ** (0.001 <*P *≤ 0.01); *** (*P *≤ 0.001)

^‡ ^a superscripted letter indicates birth weight incorporated as a linear (a) or quadratic (b) function in final model

Although the factors that significantly influenced lamb survival differed amongst breeds and depended on whether or not birth weight was included in the model (Table 2), the effect of each factor, where significant, was consistent across breeds. The risk of mortality decreased with birth weight; however, for some breeds an increase in risk was also identified for very large lambs (Table 2; see also additional file 1). Lambs from larger litters were at greater risk than those from smaller litters. Males were at greater risk than females, while the risk of mortality was highest for lambs born to younger dams (≤ 2 years old).

### Reason for death

**Figure 1:**
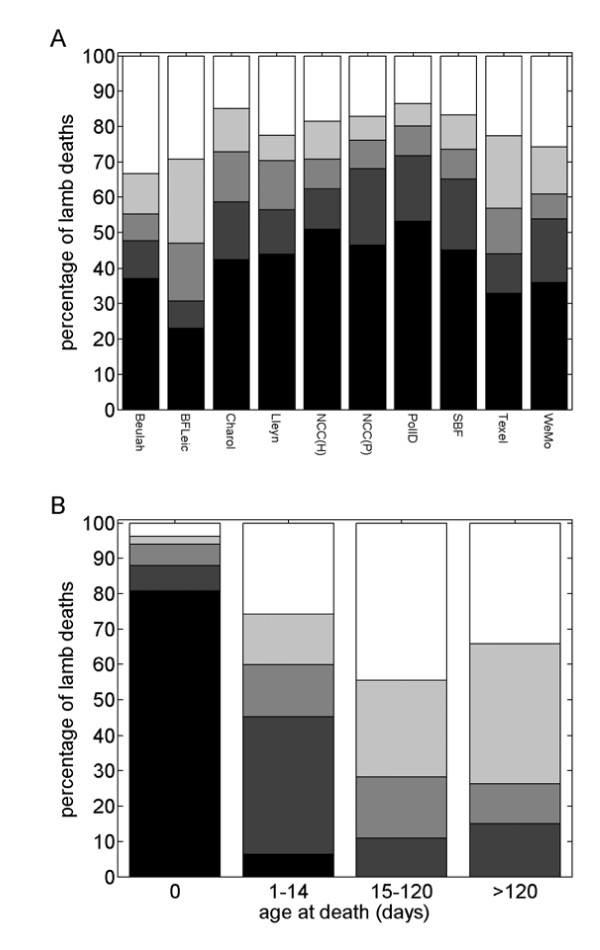
**Dependence of reasons for lamb deaths on sheep breed and age at death**. (A) Percentage of lamb deaths which were classified as lambing-associated (black), misfortune (dark grey), physical (mid-grey), disease (light grey) or other (white), and their dependence on sheep breed. Breeds are: Beulah Speckled Face (Beulah); Bluefaced Leicester (BFLeic); Charollais (Charol); Lleyn; North Country Cheviot (Hill) (NCC(H)); North Country Cheviot (Park) (NCC(P)); Poll Dorset (PollD); Scottish Blackface (SBF); Texel; and Welsh Mountain (WeMo). (B) Percentage of lamb deaths which were classified as lambing-associated (black), misfortune (dark grey), physical (mid-grey), disease (light grey) or other (white), and their dependence on age at death.

A significant interaction between breed and the remaining four factors was identified in the multinomial log-linear model; subsequent analyses were performed separately for each breed (cf. Figure 1a). For all breeds reasons for death changed significantly with age (Table 3; Figure 1b). Most lambs that died at birth did so for reasons associated with lambing (e.g. stillborn, malpresentation), while those that died at between one and 14 days old were most likely to die as a result of misfortune (e.g. taken by a predator or drowned). Lambs that died at older ages were more likely to do so for physical causes (e.g. mineral deficiencies; 15–120 days) or disease (>120 days). In addition, there were significant differences in reasons for death between years for the Scottish Blackface, North Country Cheviot (Park), Welsh Mountain and Texel breeds (Table 3), that is, mainly in extensively reared breeds. Finally, comparing lambing-related deaths with those due to misfortune in the Scottish Blackface breed showed that male lambs were less likely than females to die due to misfortune (odds ratio (OR): 0.53; 95% CI: 0.33–0.83). Importantly, there were no significant associations between reason for death and PrP genotype for any of the breeds (Table 3).

**Table 3:** Summary of factors influencing the reason for lamb deaths in ten sheep breeds in GB.

	factor^†^			
	
breed	PrP genotype	sex	age at death	year of birth
Beulah Speckled Face	ns	ns	***	ns
Bluefaced Leicester	ns	ns	***	ns
Charollais	ns	ns	***	ns
Lleyn	ns	ns	***	ns
North Country Cheviot (Hill)	ns	ns	***	ns
North Country Cheviot (Park)	ns	ns	***	***
Poll Dorset	ns	ns	***	ns
Scottish Blackface	ns	*	***	**
Texel	ns	ns	***	*
Welsh Mountain	ns	ns	***	**

^† ^significance in final model or when deleted from the model: ns (*P *> 0.05); * (0.01 <*P *≤ 0.05); ** (0.001 <*P *≤ 0.01); *** (*P *≤ 0.001)

## Discussion

Lamb survival is a complex trait and is influenced by many factors, including birth weight [25-29], litter size [19-21,26,29], sex [19-21,26-28], and age of dam [19-21,26]. Similar effects were identified in the present study for each of these factors, though not all of the individual factors were significant for all ten breeds.

The particular focus of this paper, however, was to investigate associations between PrP genotype and lamb survival, in order to help assess whether there could be deleterious effects of breeding programmes based on PrP genotype selection. A significant association was identified for the Charollais breed, with an increased risk of mortality for lambs carrying the ARR/VRQ genotype compared with those carrying the ARR/ARR genotype. However, the ARR/VRQ genotype is actively selected against in the NSP [30]; its frequency in ram lambs of the Charollais breed sampled as part of the NSP is low and decreased from 4.0% in 2002 to 2.6% in 2004 [31]. Hence, it is unlikely that this finding has any important consequences for selective breeding programmes, in general, or the NSP, in particular.

A recent study of lamb survival in two Scottish Blackface flocks found that lambs carrying the ARQ allele had higher postnatal survival than those carrying the ARR or AHQ allele [16]. A similar pattern was observed for Scottish Blackface lambs in the present analysis. However, this result was not statistically significant (*P *= 0.60), and it was not replicated in the model including birth weight. Indeed no significant associations between PrP genotype and lamb survival were identified for any of the remaining breeds considered in the study, nor were there any consistent patterns in the effect of PrP genotype on lamb survival across breeds. This lack of consistency between studies suggests that if there is an association between PrP genotype and lamb survival it may be due to linkage disequilibrium rather that pleiotropy, or may not be constant across flocks.

Although only one significant association between PrP genotype and survival was identified, not all PrP genotypes commonly found in each breed were represented in this study (see additional file 1). This reflects the fact that all participating flocks were members of the NSP and, hence, would have been selecting based on PrP genotype and, in particular, against the VRQ allele, possibly for several years prior to recruitment [31]. Consequently, most lambs in the study carried at least one ARR allele, while very few carried the VRQ allele. Indeed, almost all lambs carrying the VRQ allele were of the ARR/VRQ genotype, though some were also of the ARQ/VRQ genotype. There were, however, no ARH/VRQ, AHQ/VRQ or VRQ/VRQ carriers and, accordingly, the study could not have identified any associations between lamb survival and these PrP genotypes.

Biases could be introduced into the data, in terms of factors influencing relative differences in mortality, if selection of live lambs had occurred prior to PrP genotyping. However, the lamb crops on most farms were blood sampled before replacements were selected or lambs were sent for sale or slaughter. Because not every lamb which died on farm will have been detected in the study, biases may also arise if PrP genotype was associated with reasons for death that resulted in dead lambs not being found. It is difficult to assess the impact of this potential bias, though the present study did not identify any associations between the reason for lamb death and PrP genotype. Finally, a high proportion of records in the study were right-censored (i.e. were for lambs surviving beyond 120 days). This could reduce the power of the data-set or the precision of the results, but reflects the level of mortality in the study flocks.

Birth weight has often been identified as an important factor for lamb survival [25-29], including in the present study (Table 2). However, an appreciable number of records were missing values for this factor. When birth weight was not included in the analysis, there were changes in the factors that were significantly associated with lamb survival. In particular, litter size and age of dam were often only included in the final models excluding birth weight (Table 2), most likely reflecting associations between birth weight and these factors [14,25,29,32]. The one significant association between PrP genotype and lamb survival was identified only when birth weight was not included in the analysis. This was due to the fact that there were no birth weight records available for the dead ARR/VRQ lambs, rather than due to an association between PrP genotype and birth weight. Indeed, few significant associations with PrP and birth weight have been identified [10,14,32].

No associations between PrP genotype and reason for death were identified in this study (Table 3). The principal factor influencing the reason for death was the age at which the lamb died. Although previous studies also found that the cause of death in lambs changed with age [33-35], differences in classifications preclude direct comparison amongst studies.

## Conclusion

This study found no evidence to suggest that a selective breeding programme based on PrP genotype will have a detrimental effect on lamb survival, at least for the ten breeds included. Indeed, the only significant effect of PrP genotype identified here is likely to be of little consequence, because it found an increased risk of mortality in a genotype (ARR/VRQ) of low frequency that is actively selected against as part of current breeding strategies.

## Authors' contributions

SG performed the statistical analysis and drafted the manuscript. CC managed and collated the final data-sets and performed the statistical analysis. KH provided input to the statistical analysis. KB matched NSP genotypes to Signet records and CD managed the Signet data. ET and WH managed the collection of data on dead lambs. WH, SCB, BV and RE conceived the study and led the project. All authors critically revised the manuscript and read and approved the final version.

## Supplementary Material

Additional File 1 **Additional material.** Click here for file
