# Supplementary material for: Associations between lamb survival and prion protein genotype: analysis of data for ten sheep breeds in Great Britain
Source: BMC Vet Res. 2009 Jan 21;5:3. doi: 10.1186/1746-6148-5-3 (PMC2637852; doi:10.1186/1746-6148-5-3)
Supplement: Additional File 1 — Additional material. [file 1746-6148-5-3-S1.doc]

**Additional Material** ***BMC Veterinary Research***

**Associations between lamb survival and prion protein genotype: analysis of data for ten sheep breeds in Great Britain**

Simon Gubbins^1^, Charlotte J. Cook^2^, Kieran Hyder^2^, Kay Boulton^3^, Carol Davis^3^, Eurion Thomas^4^, Will Haresign^5^, Stephen C. Bishop^6^, Beatriz Villanueva^7^ & Rachel D. Eglin^2,*^

^1^ Institute for Animal Health, Pirbright Laboratory, Ash Road, Pirbright, Surrey GU24 0NF, U.K.

^2^ Centre for Epidemiology and Risk Analysis, Veterinary Laboratories Agency, Woodham Lane, New Haw, Addlestone, Surrey KT15 3NB, U.K.

^3^ Meat and Livestock Commission, Winterhill House, Snowdon Drive, Milton Keynes MK6 1AX, U.K.

^4^ Innovis Ltd, Peithyll Centre, Capel Dewi, Aberystwyth, Ceredigon SY23 3HU, U.K.

^5^ Institute of Biological, Environmental and Rural Sciences, Aberystwyth University, Llanbadarn Campus, Aberystwyth, Ceredigon SY23 3AL, U.K.

^6^ The Roslin Institute and R(D)SVS, University of Edinburgh, Roslin BioCentre, Roslin, Midlothian EH25 9PS, U.K.

^7^ Scottish Agricultural College, West Mains Road, Edinburgh EH9 3JG, U.K.

This additional material provides full details of the prion protein (PrP) genotypes included in the study for each breed (Table S.1); and the final models for lamb survival times for each breed for the analyses including (Table S.2) or excluding (Table S.3) birth weight as a factor.

**References**

Dawson, M., Hoinville, L.J., Hosie, B.D. & Hunter, N. 1998 Guidance on the use of PrP genotyping as an aid to the control of clinical scrapie. *Vet. Record* **142** 623-625.

Eglin, R.D., Warner, R., Gubbins, S., Sivam, S.K. & Dawson, M. 2005 Frequencies of prion protein (PrP) genotypes in thirty eight sheep breeds sampled in the National Scrapie Plan for Great Britain. *Vet. Record* **156**, 433-437.

*Table S.1. Numbers of dead/live lambs of each prion protein (PrP) genotype included in the study of lamb survival in ten sheep breeds in Great Britain.*

| PrP genotype | Beulah Speckled Face | Bluefaced Leicester | Charollais | Lleyn | North County Cheviot (Hill) | North County Cheviot (Park) | Poll Dorset | Scottish Blackface | Texel | Welsh Mountain |
| --- | --- | --- | --- | --- | --- | --- | --- | --- | --- | --- |
| ARR/ARR | 37/1017 | 56/395 | 424/4395 | 124/2251 | 49/641 | 63/936 | 169/3190 | 132/1066 | 177/4282 | 50/1057 |
| ARR/AHQ | 1/72 | 19/156 | -^†^ | 24/335 | 15/329 | 35/235 | 1/8^†^ | 44/267 | 14/247 | 23/430 |
| ARR/ARH | -^†^ | -^†^ | -^†^ | 4/386 | -^†^ | -^†^ | -^†^ | -^†^ | 141/3270 | -^†^ |
| ARR/ARQ | 31/572 | 9/119 | 87/1257 | 23/483 | 68/718 | 94/847 | 34/953 | 216/2018 | 38/1079 | 20/717 |
| AHQ/AHQ | - | - | -^†^ | - | 2/34 | - | -^†^ | 3/14 | - | 1/8 |
| AHQ/ARH | -^†^ | -^†^ | -^†^ | 1/12 | -^†^ | -^†^ | -^†^ | -^†^ | - | -^†^ |
| AHQ/ARQ | - | 1/8 | -^†^ | - | 8/138 | 16/71 | -^†^ | 27/137 | - | 2/37 |
| ARH/ARH | -^†^ | -^†^ | -^†^ | - | -^†^ | -^†^ | -^†^ | -^†^ | 19/251 | -^†^ |
| ARH/ARQ | -^†^ | -^†^ | -^†^ | - | -^†^ | -^†^ | -^†^ | -^†^ | 8/91 | -^†^ |
| ARQ/ARQ | 2/74 | - | - | - | 14/119 | 13/132 | 4/75 | 45/521 | - | 2/39 |
| ARR/VRQ | - | -^†^ | 9/69 | 2/8 | 1/35 | 1/34 | 7/265 | 7/57 | 4/73 | - |
| AHQ/VRQ | - | -^†^ | -^†^ | - | - | - | -^†^ | - | - | - |
| ARH/VRQ | -^†^ | -^†^ | -^†^ | - | -^†^ | -^†^ | -^†^ | -^†^ | - | -^†^ |
| ARQ/VRQ | - | -^†^ | - | - | 1/16 | - | - | 5/18 | - | - |
| VRQ/VRQ | - | -^†^ | - | - | - | - | - | - | - | - |

^†^ PrP genotype not commonly found in breed (see Dawson *et al*. 1998; Eglin *et al*. 2005)

Table S.2. *Hazard ratios (and 95% confidence intervals) in the final models for lamb survival in ten sheep breeds in Great Britain (including birth weight)*.

| factor | Beulah Speckled Face | Bluefaced Leicester | Charollais | Lleyn | North County Cheviot (Hill) | North County Cheviot (Park) |
| --- | --- | --- | --- | --- | --- | --- |
| birth weight |  |  |  |  |  |  |
| linear | 0.35 (0.23, 0.53) | 0.72 (0.58, 0.89) | 0.14 (0.09, 0.21) | 0.52 (0.44, 0.60) | 0.52 (0.42, 0.64) | 0.22 (0.11, 0.42) |
| quadratic | - | - | 1.17 (1.13, 1.22) | - | - | 1.12 (1.05, 1.18) |
| litter size |  |  |  |  |  |  |
| 1 | - | - | baseline | - | baseline | - |
| 2 | - | - | 0.67 (0.52, 0.86) | - | 0.55 (0.34, 0.90) | - |
| ≥3 | - | - | 0.79 (0.58, 1.09) | - | 0.44 (0.14, 1.39) | - |
| sex |  |  |  |  |  |  |
| female | baseline | - | - | baseline | baseline | baseline |
| male | 4.35 (2.28, 8.30) | - | - | 3.82 (2.74, 5.33) | 3.74 (2.34, 5.99) | 2.92 (1.97, 4.31) |
| age of dam |  |  |  |  |  |  |
| ≤2 years old | - | - | - | - | - | - |
| 3 years old | - | - | - | - | - | - |
| 4 years old | - | - | - | - | - | - |
| 5 years old | - | - | - | - | - | - |
| ≥6 years old | - | - | - | - | - | - |
| year of birth |  |  |  |  |  |  |
| 2004 | baseline | baseline | baseline | baseline | baseline | baseline |
| 2005 | 0.44 (0.22, 0.88) | 1.02 (0.59, 1.77) | 1.54 (1.22, 1.94) | 0.75 (0.53, 1.06) | 0.21 (0.12, 0.38) | 0.58 (0.37, 0.89) |
| 2006 | 0.22 (0.08, 0.58) | 0.30 (0.14, 0.63) | 1.68 (1.25, 2.26) | 0.25 (0.15, 0.43) | 0.14 (0.05, 0.34) | 0.06 (0.02, 0.14) |

Table S.2 (continued).

| factor | Poll Dorset | Scottish Blackface | Texel | Welsh Mountain |
| --- | --- | --- | --- | --- |
| birth weight |  |  |  |  |
| linear | 0.12 (0.07, 0.19) | 0.14 (0.08, 0.26) | 0.77 (0.68, 0.86) | 0.25 (0.16, 0.37) |
| quadratic | 1.20 (1.14, 1.28) | 1.22 (1.12, 1.32) | - | - |
| litter size |  |  |  |  |
| 1 | baseline | baseline | - | - |
| 2 | 0.63 (0.43, 0.94) | 0.81 (0.62, 1.07) | - | - |
| ≥3 | 0.86 (0.52, 1.44) | 1.43 (0.86, 2.38) | - | - |
| sex |  |  |  |  |
| female | baseline | baseline | baseline | baseline |
| male | 2.50 (1.79, 3.50) | 2.88 (2.23, 3.72) | 1.55 (1.20, 2.00) | 6.61 (3.89, 11.2) |
| age of dam |  |  |  |  |
| ≤2 years old | - | baseline | baseline | baseline |
| 3 years old | - | 0.65 (0.46, 0.90) | 0.85 (0.62, 1.16) | 0.34 (0.16, 0.70) |
| 4 years old | - | 0.54 (0.38, 0.78) | 0.84 (0.57, 1.24) | 0.39 (0.19, 0.79) |
| 5 years old | - | 0.74 (0.52, 1.05) | 1.12 (0.73, 1.73) | 0.32 (0.13, 0.79) |
| ≥6 years old | - | 0.69 (0.43, 1.10) | 1.82 (1.18, 2.80) | 0.35 (0.13, 0.91) |
| year of birth |  |  |  |  |
| 2004 | baseline | baseline | - | baseline |
| 2005 | 1.16 (0.81, 1.65) | 1.59 (1.19, 2.14) | - | 2.63 (1.25, 5.55) |
| 2006 | 0.50 (0.31, 0.80) | 0.33 (0.23, 0.46) | - | 0.61 (0.29, 1.30) |

Table S.3. *Hazard ratios (and 95% confidence intervals) in the final models for lamb survival in ten sheep breeds in Great Britain (excluding birth weight)*.

| factor | Beulah Speckled Face | Bluefaced Leicester | Charollais | Lleyn | North County Cheviot (Hill) | North County Cheviot (Park) |
| --- | --- | --- | --- | --- | --- | --- |
| PrP genotype |  |  |  |  |  |  |
| ARR/ARR | - | - | baseline | - | - | - |
| ARR/ARQ | - | - | 0.90 (0.71, 1.15) | - | - | - |
| ARR/VRQ | - | - | 2.67 (1.31, 5.44) | - | - | - |
| litter size |  |  |  |  |  |  |
| 1 | baseline | - | baseline | baseline | baseline | baseline |
| 2 | 2.63 (1.18, 5.89) | - | 0.97 (0.78, 1.20) | 1.40 (0.94, 2.07) | 1.55 (1.09, 2.20) | 1.37 (0.93, 2.03) |
| ≥3 | 10.2 (4.17, 25.3) | - | 1.82 (1.41, 2.35) | 1.80 (1.10, 2.94) | 3.70 (1.83, 7.50) | 3.09 (1.93, 4.95) |
| sex |  |  |  |  |  |  |
| female | baseline | - | - | baseline | baseline | baseline |
| male | 8.93 (4.96, 16.0) | - | - | 2.91 (2.13, 3.98) | 2.50 (1.76, 3.57) | 2.33 (1.77, 3.07) |
| age of dam |  |  |  |  |  |  |
| ≤2 years old | - | baseline | - | baseline | - | baseline |
| 3 years old | - | 0.66 (0.37, 1.17) | - | 0.75 (0.50, 1.14) | - | 0.51 (0.34, 0.75) |
| 4 years old | - | 0.64 (0.35, 1.18) | - | 0.71 (0.44, 1.15) | - | 0.81 (0.55, 1.17) |
| 5 years old | - | 0.31 (0.11, 0.89) | - | 0.35 (0.18, 0.67) | - | 0.56 (0.35, 0.90) |
| ≥6 years old | - | 1.43 (0.68, 3.01) | - | 0.61 (0.38, 0.99) | - | 0.74 (0.48, 1.15) |
| year of birth |  |  |  |  |  |  |
| 2004 | - | baseline | baseline | baseline | baseline | - |
| 2005 | - | 0.81 (0.46, 1.40) | 1.66 (1.34, 2.06) | 0.72 (0.52, 1.01) | 0.41 (0.27, 0.64) | - |
| 2006 | - | 0.43 (0.23, 0.79) | 1.89 (1.46, 2.44) | 0.29 (0.18, 0.48) | 1.30 (0.78, 2.18) | - |

Table S.3 (continued).

| factor | Poll Dorset | Scottish Blackface | Texel | Welsh Mountain |
| --- | --- | --- | --- | --- |
| PrP genotype |  |  |  |  |
| ARR/ARR | - | - | - | - |
| ARR/ARQ | - | - | - | - |
| ARR/VRQ | - | - | - | - |
| litter size |  |  |  |  |
| 1 | baseline | baseline | baseline | baseline |
| 2 | 0.95 (0.68, 1.32) | 1.29 (1.04, 1.60) | 1.15 (0.89, 1.48) | 1.84 (1.19, 2.83) |
| ≥3 | 2.09 (1.36, 3.22) | 5.31 (3.66, 7.70) | 1.78 (1.27, 2.49) | 4.43 (1.48, 13.2) |
| sex |  |  |  |  |
| female | baseline | baseline | baseline | baseline |
| male | 2.22 (1.65, 2.98) | 2.45 (2.02, 2.99) | 1.40 (1.15, 1.70) | 5.10 (3.38, 7.70) |
| age of dam |  |  |  |  |
| ≤2 years old | baseline | baseline | baseline | baseline |
| 3 years old | 0.51 (0.33, 0.79) | 0.57 (0.44, 0.73) | 0.78 (0.60, 1.01) | 0.43 (0.23, 0.79) |
| 4 years old | 0.61 (0.41, 0.91) | 0.50 (0.38, 0.65) | 0.61 (0.45, 0.84) | 0.32 (0.17, 0.61) |
| 5 years old | 1.03 (0.69, 1.52) | 0.67 (0.50, 0.88) | 1.06 (0.76, 1.48) | 0.40 (0.20, 0.78) |
| ≥6 years old | 0.64 (0.41, 0.99) | 0.55 (0.39, 0.79) | 1.25 (0.88, 1.79) | 0.74 (0.39, 1.38) |
| year of birth |  |  |  |  |
| 2004 | baseline | baseline | baseline | - |
| 2005 | 1.28 (0.95, 1.72) | 1.71 (1.35, 2.17) | 1.03 (0.79, 1.32) | - |
| 2006 | 0.47 (0.31, 0.72) | 0.54 (0.42, 0.70) | 1.46 (1.13, 1.88) | - |
